# Anisotropic Cotton-Stalk-Derived Hydrothermally Treated Cellulose–Chitosan Aerogels Toward Anionic Dye Adsorption and Water-in-Oil Emulsion Separation

**DOI:** 10.3390/gels12090814

**Published:** 2026-09-05

**Authors:** Shixue He, Chengbo Zhang, Daning Lang, Ronglan Wu

**Affiliations:** 1 Key Laboratory of Oil & Gas Fine Chemicals, Ministry of Education & Xinjiang Uygur Autonomous Region, School of Chemical Engineering, Xinjiang University, Urumqi 830017, China; 2 School of Mechanical Engineering & Center for Post-doctoral Studies of Mechanical Engineering, Xinjiang University, Urumqi 830017, China

**Keywords:** cotton stalk, cellulose aerogel, chitosan, Congo red adsorption, oil/water separation, water-in-oil emulsion

## Abstract

Transforming agricultural residues into functional porous materials provides a sustainable strategy for wastewater remediation. Herein, cellulose was separated from cotton stalks via formic acid-assisted hemicellulose extraction and sodium chlorite delignification, and then sulfuric acid hydrolysis. Chitosan-assisted hydrothermally treated cellulose (CC) was prepared via hydrothermal treatment in the presence of chitosan. Anisotropic CC/chitosan composite aerogels were prepared via glutaraldehyde crosslinking and unidirectional freeze-drying. The hydrophilic CC/CS aerogel exhibited an oriented porous structure, a low density of 0.03 g cm^−3^, and a porosity of 85.33%. For Congo red (CR) adsorption, the equilibrium data were described well by the pseudo-second-order kinetic and Langmuir isotherm models, with a calculated maximum adsorption capacity of 483.09 mg g^−1^. Electrostatic attraction, hydrogen bonding, and pore-mediated retention jointly contributed to CR uptake. To realize oil–water separation, methyltrimethoxysilane (MTMS) vapor modification was applied to prepare hydrophobic aerogel (M-CC/CS). M-CC/CS presented an initial water contact angle (WCA) of around 134°, and the WCA remained above 115° after 600 s of water droplet exposure. The aerogel showed absorption capacities of 16.22–40.13 g g^−1^ toward various oils and organic solvents. Under gravity, M-CC/CS separated immiscible oil/water mixtures at a flux of 565.47 L m^−2^ h^−1^ and several water-in-oil (W/O) emulsions with efficiencies above 99.9% while maintaining high separation efficiency over 10 cycles. This work demonstrates a cotton-stalk-derived aerogel platform whose hydrophilic and hydrophobically modified forms can be used for dye adsorption and oily water treatment, respectively.

## 1. Introduction

Dye-containing effluents and oily wastewater are persistent environmental concerns because they contain chemically stable organic contaminants, dispersed droplets, and, in many cases, surfactants that complicate conventional treatment [1,2]. These components form stable colloidal and emulsified systems in water, which severely inhibit flocculation, precipitation and filtration in conventional treatment processes, leading to low treatment efficiency and secondary water pollution [3]. Porous separation materials have therefore attracted considerable attention because their low density, interconnected pore networks, and tunable surface chemistry enable adsorption, absorption, and gravity-driven phase separation, overcoming the technical bottlenecks of traditional treatment technologies [4,5]. Recently reported membranes, sponges, and aerogels have achieved efficient removal of dyes, free oils and stabilized emulsions [6,7]. For example, Qin et al. used cassava residue as the building block with ferroferric oxide introduced for magnetic separation. With polyethyleneimine as the functional component, a novel three-dimensionally porous cellulose-based aerogel was constructed to capture CR efficiently and selectively [8]. Via unidirectional freeze-drying and carbonization, Zhou et al. obtained a compressible, anisotropic sheet-shaped hydrophobic and oleophilic carbon aerogel composed of graphene, polyvinyl alcohol and cellulose nanofibers for selective oil–water separation [9]. However, most reported materials can only adapt to single-type wastewater treatment [10], and the simultaneous requirements of low cost, structural stability, recyclability, and sustainable feedstocks remain important challenges restricting their practical industrial application.

Natural biomass polysaccharides are promising sustainable precursors for porous materials [11]. As an abundant renewable natural polymer, cellulose possesses rich surface hydroxyl groups, enabling facile chemical modification, crosslinking assembly and effective binding with aqueous pollutant molecules through hydrogen bonding and electrostatic interactions [12,13]. Chitosan contains abundant active amino groups and can interact with cellulose mainly through hydrogen bonding and other intermolecular interactions [14]. With the introduction of a chemical crosslinker, the cellulose/chitosan network can be further stabilized, improving the structural integrity and stability of the composite aerogel while retaining amino-containing sites for adsorption of anionic pollutants [15].

At present, most reported biomass porous materials only achieve a single wastewater treatment function: hydrophilic chitosan/cellulose aerogels are limited to dye adsorption [16], while hydrophobic carbon aerogels are only suitable for oil removal [17]. Fabricating two independent material systems separately increases raw material and manufacturing costs, which severely restricts large-scale application. Cotton stalk is an abundant byproduct of Xinjiang cotton planting industry; massive abandoned cotton stalks cause land occupation and incineration pollution, so converting cotton stalk into dual-functional aerogel can simultaneously realize agricultural waste valorization and multi-type wastewater treatment. Xinjiang cotton stalk, a plentiful agricultural waste rich in cellulose, serves as an economical and sustainable raw material for high-value functional porous materials [18]. Pure cellulose aerogels exhibit inherent hydrophilicity, which is favorable for dye adsorption but restricts their application in oil–water separation [19]. Surface modification can reduce the surface free energy of cellulose–chitosan skeletons to endow hydrophobic–oleophilic wettability [20]. Meanwhile, unidirectional freezing creates oriented lamellar pore channels, which optimize mass transfer efficiency and enhance structural compressibility and resilience [21]. Combining a hydrophilic adsorbent with its hydrophobically modified counterpart is therefore a practical route to address different wastewater streams without requiring an entirely separate material platform. Although cellulose/chitosan aerogels, glutaraldehyde crosslinking, directional freezing, and silane hydrophobization have been widely reported, the distinction of the present work lies in integrating cotton-stalk-derived cellulose, chitosan-assisted hydrothermal treatment, anisotropic pore construction, and complementary wettability regulation within a single biomass-derived material platform.

In this study, cellulose was extracted from Xinjiang cotton stalks and converted into a CC. CC was crosslinked with chitosan and unidirectionally freeze-dried to construct an anisotropic CC/CS aerogel with an ordered pore structure and a stable crosslinking network. The adsorption behavior of hydrophilic CC/CS toward anionic CR was evaluated through pH-dependent, interference, kinetic, isotherm, and recycling tests to systematically explore its internal dye adsorption mechanism and anti-interference performance in complex water environments. The aerogel was then modified with MTMS to obtain hydrophobic M-CC/CS, whose wettability, oil absorption, immiscible oil/water separation, and W/O emulsion separation performances were investigated. Thus, rather than relying on a new individual processing technique, this study emphasizes the complementary use of hydrophilic CC/CS for dye adsorption and hydrophobic M-CC/CS for oily water treatment derived from the same cotton-stalk-based platform.

## 2. Results and Discussion

### 2.1. Morphology and Physicochemical Characterization

**Figure 1:**
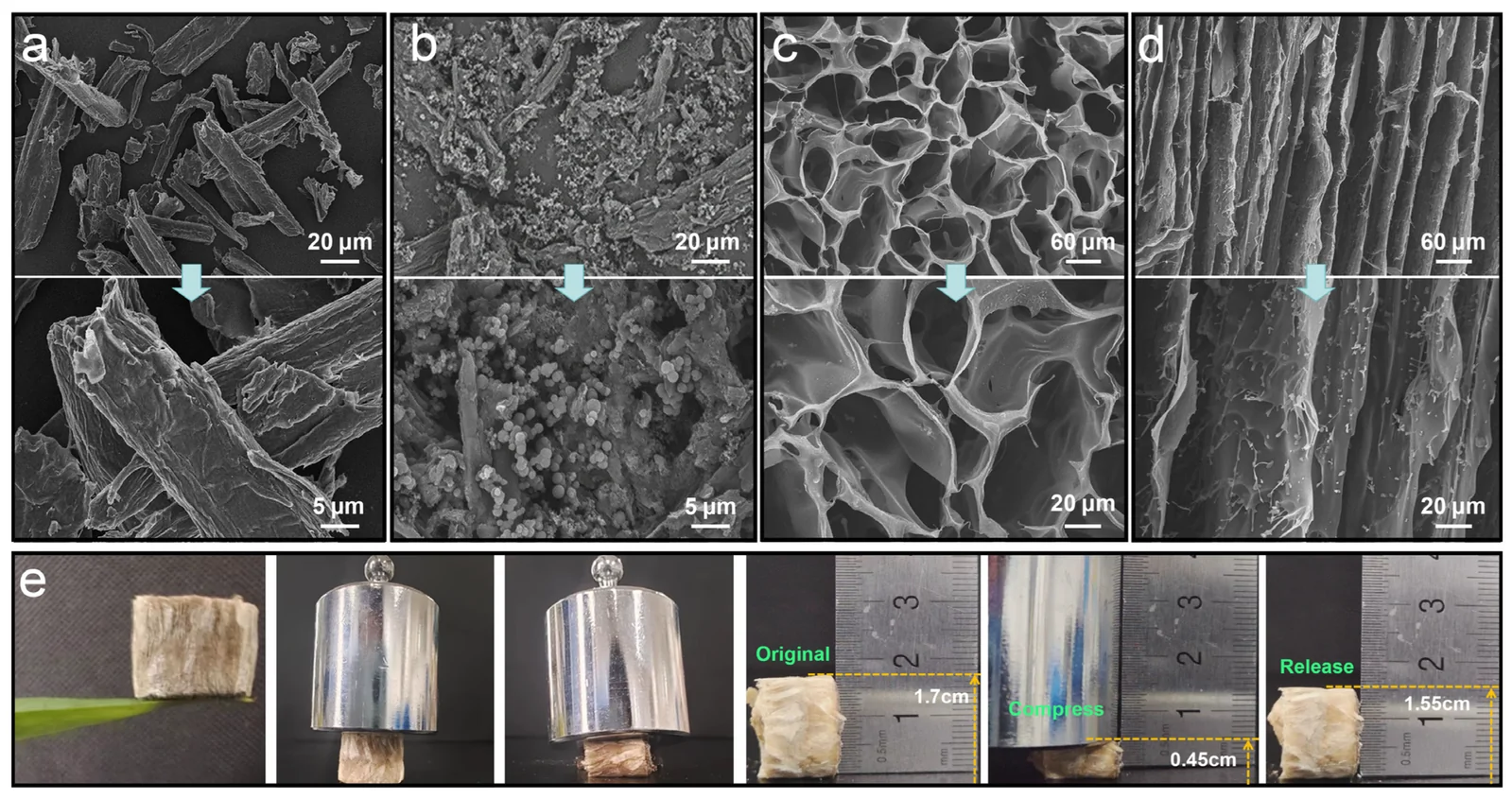
SEM images of isolated (**a**) cellulose and (**b**) CC; SEM images of the (**c**) cross-section and (**d**) longitudinal section of CC/CS; and (**e**) photographs demonstrating the low density, load-bearing ability, and compression recovery of CC/CS.

SEM images show that the isolated cellulose retained a rod-like morphology with a comparatively smooth surface (Figure 1a). After the hydrothermal treatment, CC displayed a rougher, more wrinkled surface, together with dispersed spherical domains (Figure 1b). Because chitosan was present during the hydrothermal step, these domains are attributed to the deposited carbon-rich chitosan/cellulose phase; however, their composition cannot be assigned from SEM alone. Following glutaraldehyde crosslinking and unidirectional freezing, CC/CS formed a three-dimensional anisotropic architecture. The cross-section contained an interconnected cellular network, whereas the longitudinal section exhibited aligned lamellar channels (Figure 1c,d). This morphology is consistent with ice-templated directional solidification and is expected to facilitate axial mass transport [22]. The monolithic aerogel supported a 100 g load without visible collapse and recovered after compression (Figure 1e and Video S1). After being compressed from an initial height of 1.70 cm to approximately 0.45 cm, the sample recovered to approximately 1.55 cm, corresponding to a height recovery of about 88.0%. These observations demonstrate the low density and recoverable anisotropic structure of CC/CS.

**Figure 2:**
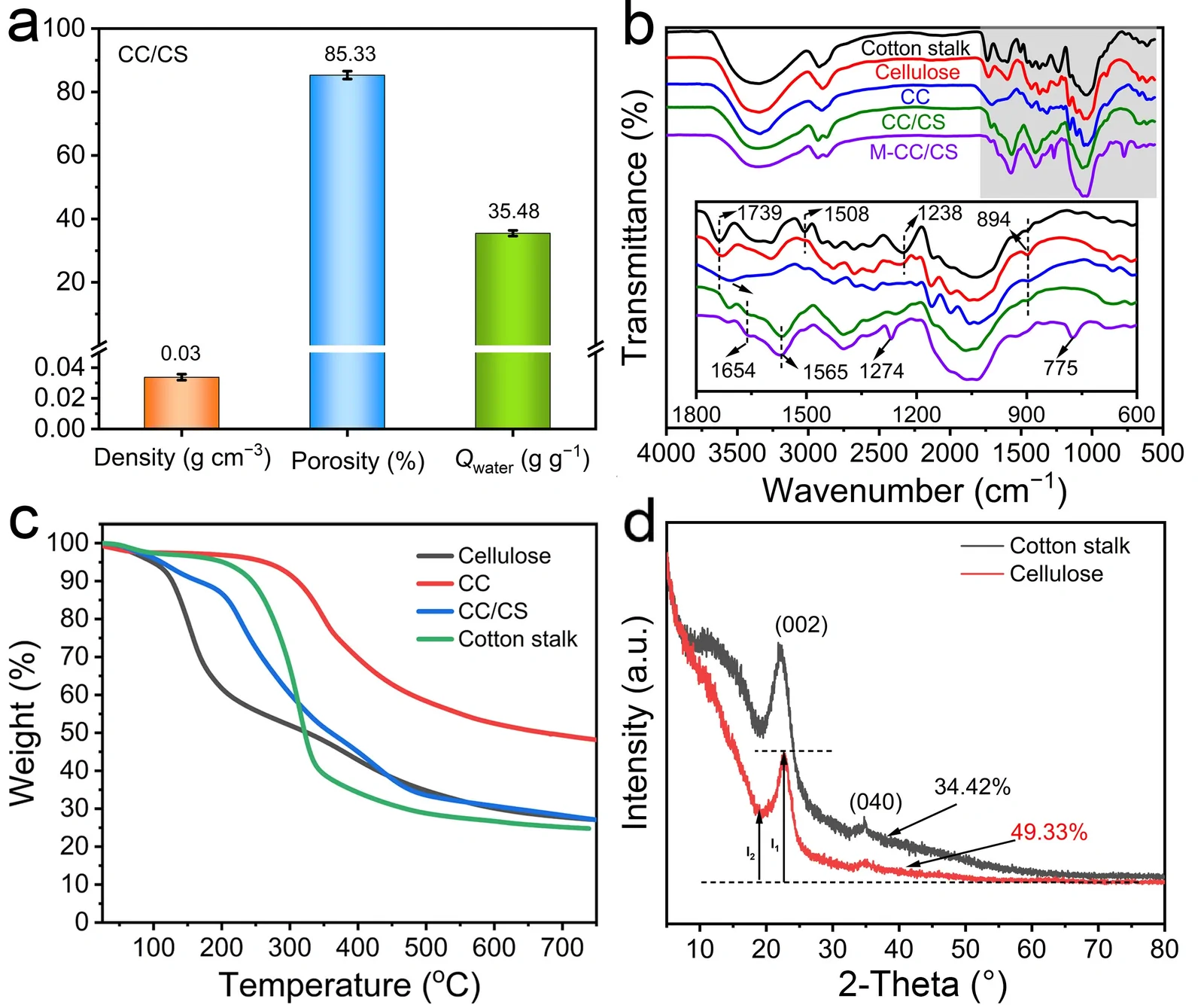
(**a**) Bulk density, porosity, and water-absorption capacity of CC/CS; (**b**) FTIR spectra; (**c**) thermogravimetric curves; and (**d**) XRD patterns of raw cotton stalk and purified cellulose.

CC/CS had a bulk density of 0.03 g cm^−3^, a porosity of 85.33%, and a water-absorption capacity of 35.48 g g^−1^ (Figure 2a). The high water uptake is consistent with the interconnected pore network and the abundant hydroxyl and amino groups in the composite. In the FTIR spectrum of the raw cotton stalk (Figure 2b), the bands at approximately 1739, 1508, and 1238 cm^−1^ are associated with carbonyl-containing hemicellulose/lignin components, aromatic skeletal vibrations of lignin, and C–O vibrations of lignin-related structures, respectively. These bands were markedly weakened after purification, whereas the cellulose-related band near 894 cm^−1^ remained evident, indicating substantial removal of hemicellulose and lignin and a relative enrichment of cellulose after purification [23]. After hydrothermal treatment, changes near 1700 cm^−1^ indicated partial dehydration and carbonization of the cellulose/chitosan precursor [24]. For CC/CS, the characteristic absorption band centered at 1654 cm^−1^ corresponds to C=N imine bonds generated by the Schiff base reaction between glutaraldehyde and amino groups of chitosan, which is consistent with previous chitosan crosslinking research. The band near 1565 cm^−1^ is associated with amino-group vibrations [25]. Because glutaraldehyde crosslinking consumes amino groups and may alter pore accessibility, the crosslinking degree can influence CR adsorption; however, the effect of GA concentration was not systematically evaluated at the fixed dosage used in the present study. After MTMS treatment, additional bands near 1274 and 775 cm^−1^ were assigned to Si–CH_3_ and Si–O–Si vibrations, supporting deposition and condensation of the silane-derived phase on CC/CS [26]. The thermogravimetric curves (Figure 2c) show that removal of hemicellulose and lignin altered the degradation profile of cotton stalk cellulose. After hydrothermal treatment, CC exhibited delayed thermal weight loss below 180 °C and higher residual char yield (48.21%) at high temperature compared with raw cotton stalk, supporting partial dehydration and structural transformation of the cellulose/chitosan precursor rather than extensive carbonization or graphitization. The XRD patterns further revealed the structural changes induced by purification (Figure 2d). The crystallinity index, calculated using CrI = (I_1_ − I_2_)/I_1_ × 100%, increased from 34.42% for the raw cotton stalk to 49.33% for the purified cellulose. Here, I_1_ represents the maximum intensity of the (002) crystalline peak, while I_2_ represents the minimum intensity at approximately 2θ = 18° associated with the amorphous contribution. This increase is attributed to the removal of amorphous hemicellulose and lignin during purification, thereby increasing the relative proportion of crystalline cellulose domains.

### 2.2. Adsorption Performance of CC/CS Toward CR

**Figure 3:**
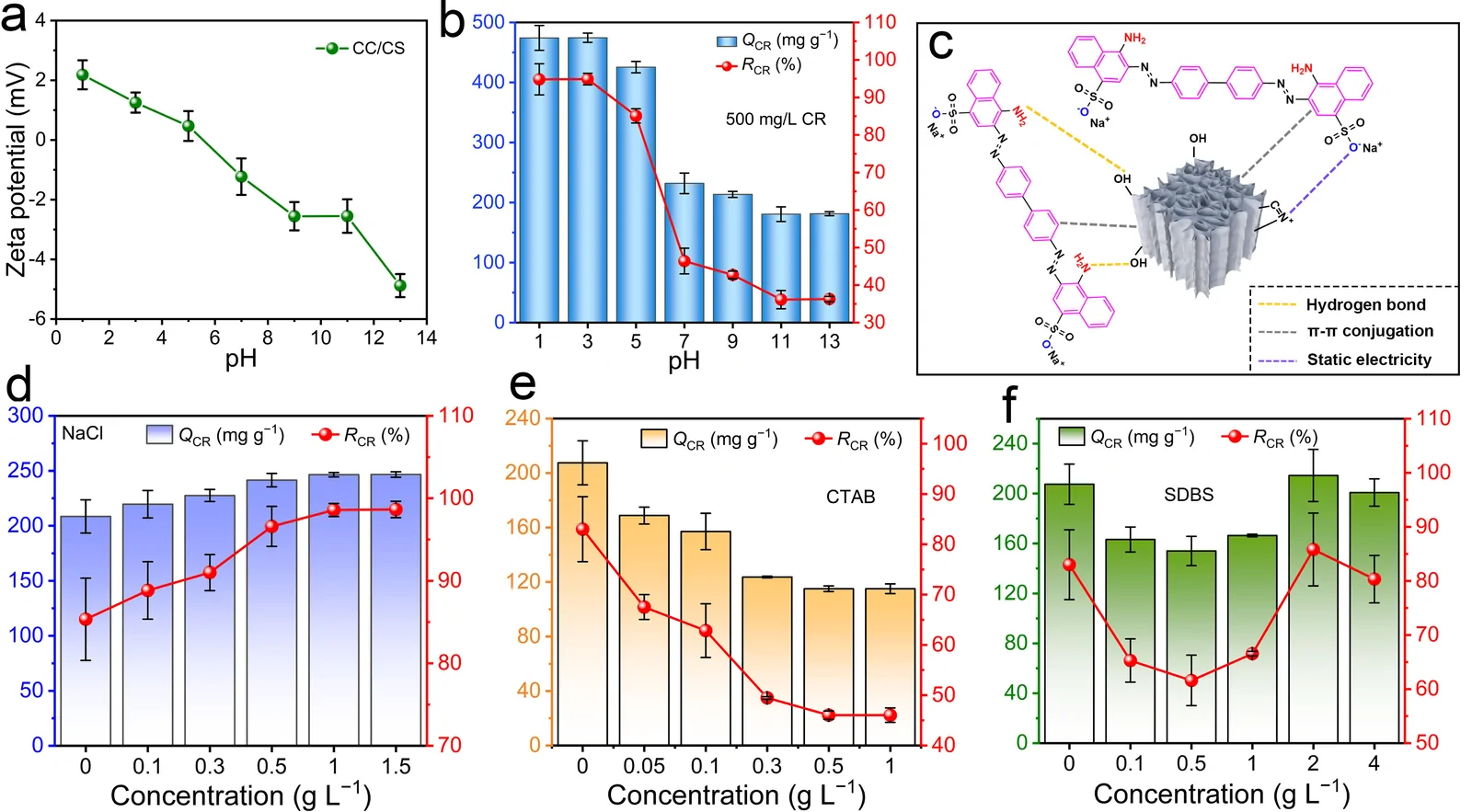
(**a**) Zeta potential and (**b**) equilibrium adsorption performance of CC/CS at different pH values; (**c**) proposed interactions between CC/CS and CR; effects of coexisting (**d**) NaCl, (**e**) CTAB, and (**f**) SDBS on CR adsorption.

The solution pH strongly affected the surface charge of CC/CS and its adsorption of CR. The zeta potential decreased with increasing pH and crossed zero at approximately pH = 5 (Figure 3a). At pH values below the isoelectric point, protonation of amino groups generated positively charged sites, whereas deprotonation at higher pH progressively reduced the positive charge and ultimately produced a negatively charged surface. Consistent with this trend, the CR adsorption capacity and removal efficiency decreased as the pH increased (Figure 3b). The favorable adsorption under acidic conditions is primarily attributed to electrostatic attraction between protonated amino groups on CC/CS and the sulfonate groups of CR, together with hydrogen bonding and pore-mediated retention.

CR is an anionic bis-azo dye containing sulfonate groups that remain negatively charged over a broad pH range. Under acidic conditions, protonated amino groups on CC/CS provide electrostatically favorable adsorption sites for CR. The hydroxyl and amino groups of CC/CS may also form hydrogen bonds with the amino, azo-associated, and sulfonate-containing moieties of CR. As the pH increases, the decline in positive surface charge and the development of electrostatic repulsion reduce dye uptake. The interconnected porous network additionally provides transport pathways and physical retention sites. The proposed adsorption process is therefore described as the combined contribution of electrostatic attraction, hydrogen bonding, and pore-assisted mass transfer rather than a single interaction mechanism (Figure 3c).

The effect of representative coexisting species is shown in Figure 3d–f. Although increasing ionic strength can suppress anionic-dye adsorption on negatively charged adsorbents, CC/CS is positively charged under acidic conditions because of protonation of its amino groups. Increasing the NaCl concentration caused the adsorption capacity to increase slightly and then approach a plateau. This behavior may result from ionic-strength-induced screening of intermolecular repulsion and changes in dye aggregation and mass transfer. By contrast, Cetyltrimethylammonium bromide (CTAB) markedly decreased CR adsorption. The cationic surfactant can associate with anionic CR and can occupy or shield adsorption sites on CC/CS, thereby reducing the amount of freely available dye and hindering pore transport. Sodium dodecylbenzenesulfonate (SDBS) produced a non-monotonic concentration-dependent response. At low SDBS concentrations, anionic SDBS competes with CR for positively charged amino sites on CC/CS, reducing adsorption capacity. When the SDBS concentration exceeds the critical micelle concentration, SDBS micelles form in solution, encapsulating partial CR molecules and altering dye mass transfer behavior inside porous channels, which alleviates competitive inhibition and partially recovers adsorption capacity. These results demonstrate that CR adsorption is sensitive to the nature of coexisting species, with CC/CS showing relatively good salt tolerance but greater susceptibility to competing surfactants. More complex ion and pollutant mixtures in actual wastewater remain to be evaluated.

**Figure 4:**
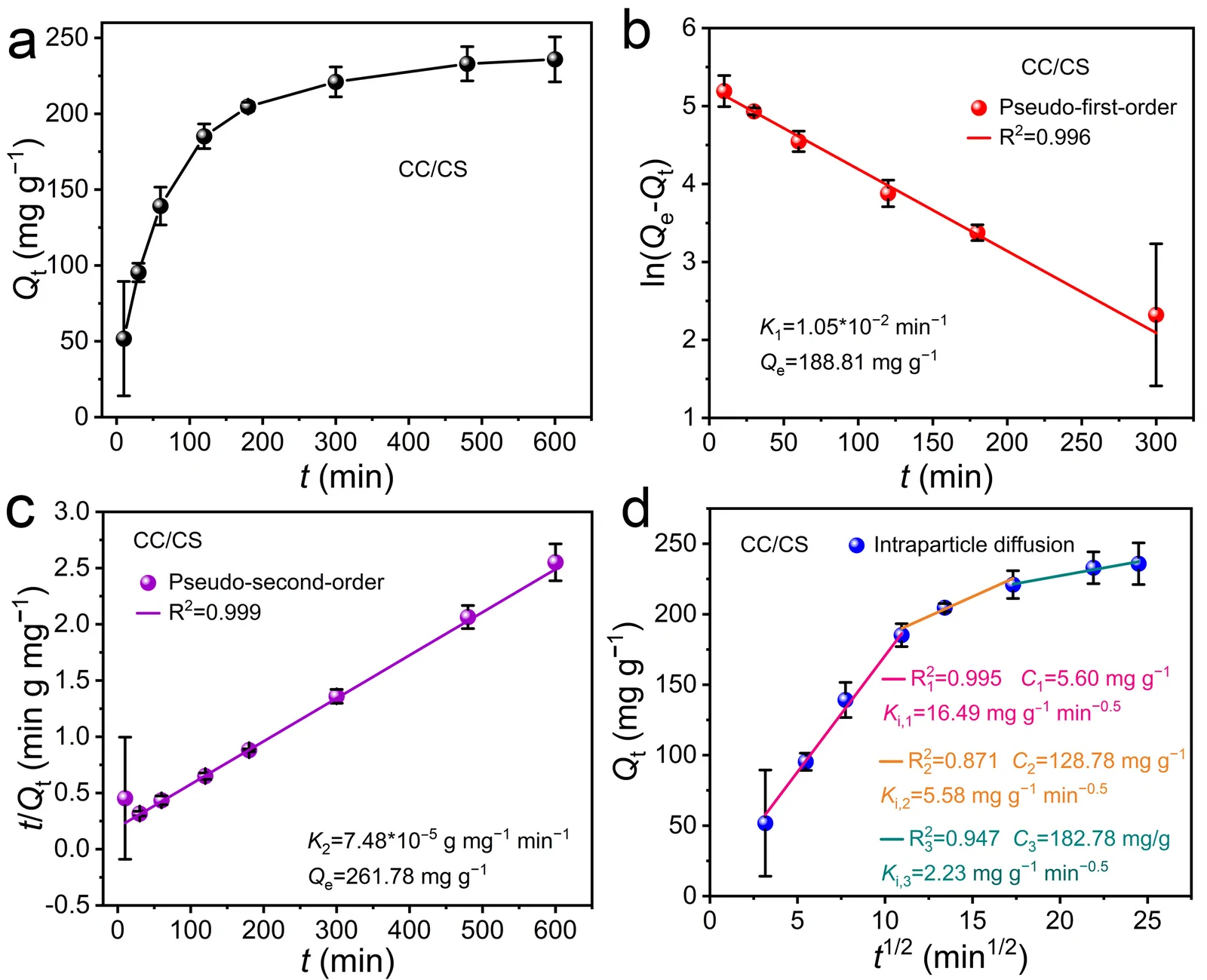
Adsorption kinetics of CR on CC/CS: (**a**) adsorption capacity as a function of contact time; fitting with the (**b**) pseudo-first-order and (**c**) pseudo-second-order kinetic models; and (**d**) intraparticle-diffusion analysis.

CR adsorption on CC/CS increased rapidly during the initial stage and subsequently approached equilibrium, reaching an experimental adsorption capacity of 235.85 mg g^−1^ at approximately 480 min (Figure 4a). The rapid initial uptake can be attributed to the relatively high concentration gradient and the availability of accessible surface and pore entry sites. As these readily accessible sites became progressively occupied, the concentration gradient decreased and transport into the internal lamellar pore network became slower, resulting in a gradual approach to equilibrium.

The pseudo-second-order model provided a better statistical description of the kinetic data than the pseudo-first-order model, and its calculated equilibrium adsorption capacity of 261.78 mg g^−1^ was reasonably close to the experimental value (Figure 4b,c). However, a better fit to the pseudo-second-order model should not be regarded by itself as proof of chemisorption or of a chemical-reaction-controlled rate. Instead, it indicates that this empirical model more adequately describes the overall time-dependent CR uptake under the investigated conditions.

The intraparticle-diffusion plot exhibited three approximately linear regions, none of which passed through the origin (Figure 4d), demonstrating that intraparticle diffusion was involved but was not the sole rate-controlling mechanism. The first region can be assigned primarily to external film transport and rapid adsorption at accessible surface sites. The second region corresponds to the slower diffusion of CR molecules into the internal pore network, whereas the third region represents the gradual attainment of adsorption equilibrium [27]. The successive decrease in the diffusion-rate constants (*K*_i1_ > *K*_i2_ > *K*_i3_) is consistent with the decreasing concentration gradient and progressive occupation of accessible sites. The nonzero and increasing intercepts indicate an appreciable boundary-layer contribution and confirm that the overall adsorption kinetics resulted from multiple coupled mass-transfer processes [28].

**Figure 5:**
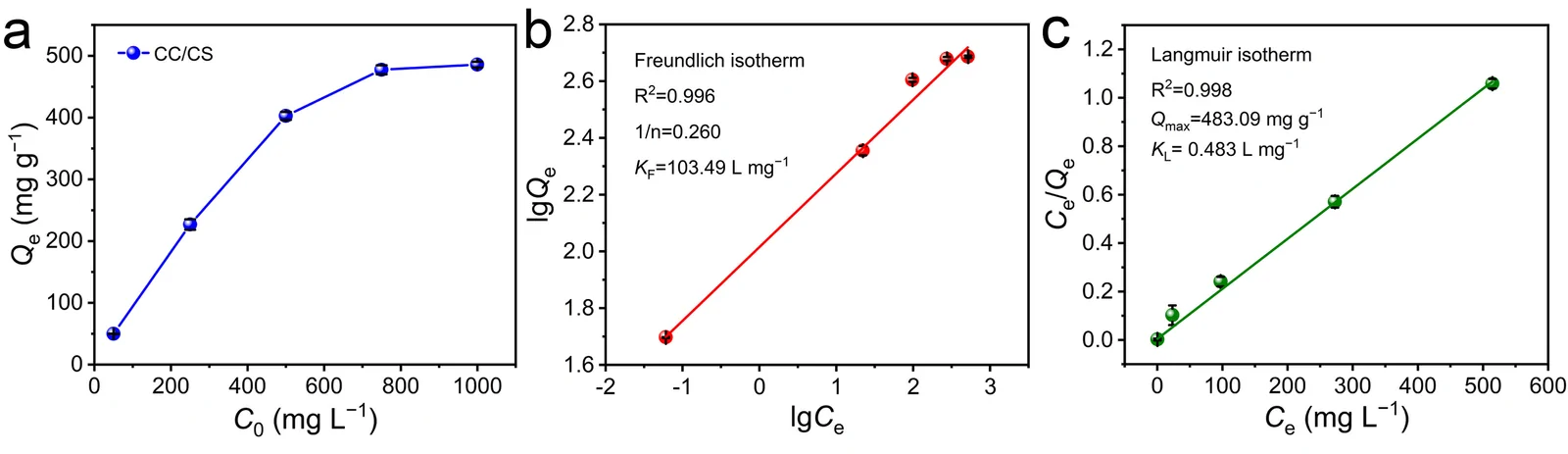
Adsorption isotherms of CR on CC/CS: (**a**) equilibrium adsorption capacity at different initial CR concentrations; (**b**) Freundlich model fitting; and (**c**) Langmuir model fitting. Experimental conditions: solution volume, 10 mL; CC/CS dosage, 10 mg; temperature, 25 °C.

The equilibrium adsorption capacity increased with the initial CR concentration and approached saturation at high concentration (Figure 5a). At an initial concentration of 1000 mg L^−1^, the experimental adsorption capacity reached 485.82 mg g^−1^. Both the Langmuir and Freundlich models fitted the data well, with R^2^ values of 0.998 and 0.996, respectively (Figure 5b,c). The slightly better Langmuir fit and the close agreement between the calculated maximum adsorption capacity (483.09 mg g^−1^) and the experimental value (485.82 mg g^−1^) suggest that adsorption on relatively homogeneous accessible sites provides a useful description of the equilibrium behavior. Meanwhile, the good Freundlich fit indicates that surface heterogeneity may also contribute to CR uptake [29]. Overall, the high adsorption capacity is attributed to the combined effects of the porous architecture and the hydroxyl- and amino-containing adsorption sites of CC/CS.

**Figure 6:**
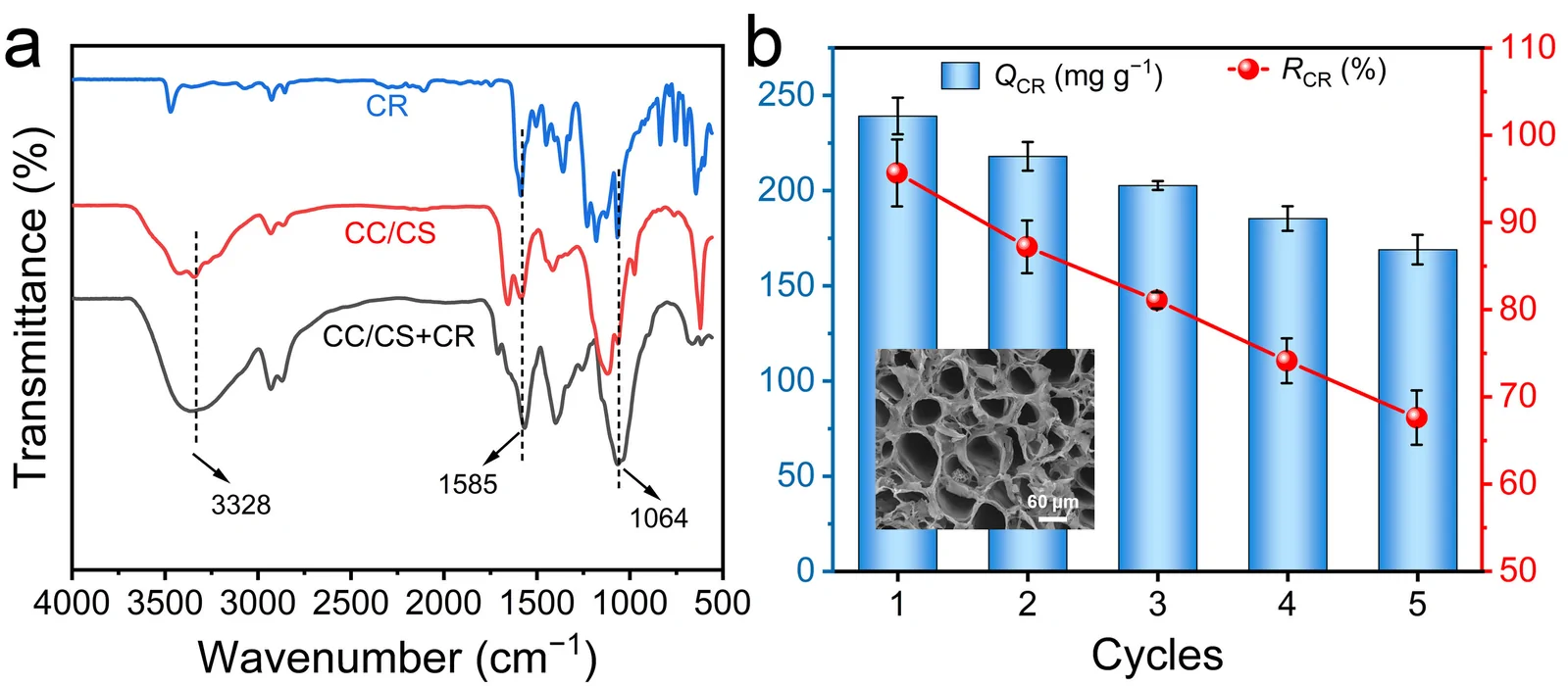
(**a**) FTIR spectra of CR, CC/CS, and CR-loaded CC/CS; (**b**) adsorption capacity and CR removal efficiency of CC/CS over five adsorption–desorption cycles using a 100 mg L^−1^ CR solution. (The inset in Figure 6b shows the SEM of the aerogel after adsorbing CR.)

FTIR spectra collected before and after adsorption provide additional evidence for interactions between CC/CS and CR (Figure 6a). After CR uptake, the broad band near 3300 cm^−1^ changed in intensity, indicating perturbation of hydroxyl- and amino-group environments. The amino characteristic band near 1565 cm^−1^ (attributed to –NH_2_) also exhibited changes, suggesting the involvement of amino groups in CR adsorption. This phenomenon mainly arises from hydrogen bonding and electrostatic interactions formed between the adsorbent and CR molecules. This band overlaps with the CR-related vibrational peak near 1585 cm^−1^ assigned to the N=N bond [30]. New or intensified bands near 1064 and 975 cm^−1^ were consistent with sulfonate- and aromatic-associated vibrations of adsorbed CR [31]. SEM observation after adsorption showed that the interconnected porous framework of CC/CS was largely preserved, while the pore walls became relatively rough and irregular (inset of Figure 6b). The open pore structure remained clearly visible, providing accessible pathways and internal surfaces for CR transport and retention. Combined with the pH-dependent zeta-potential and FTIR results, these observations indicate that CR adsorption involves electrostatic attraction between protonated amino groups and the negatively charged sulfonate groups of CR, hydrogen-bonding interactions involving hydroxyl and amino groups, and pore-assisted mass transfer and retention. The adsorption performance decreased during repeated use (Figure 6b). The CR removal efficiency declined from 95.67% in the first cycle to 67.6% after five cycles, while the adsorption capacity remained 169.11 mg g^−1^ in the fifth cycle. The decay is mainly attributed to incomplete desorption of CR molecules occupying inner pore sites, partial collapse of thin lamellar channels after repeated soaking, and gradual coverage of active amino sites by residual CR.

### 2.3. Wettability and Oil-Absorption Performance of M-CC/CS

**Figure 7:**
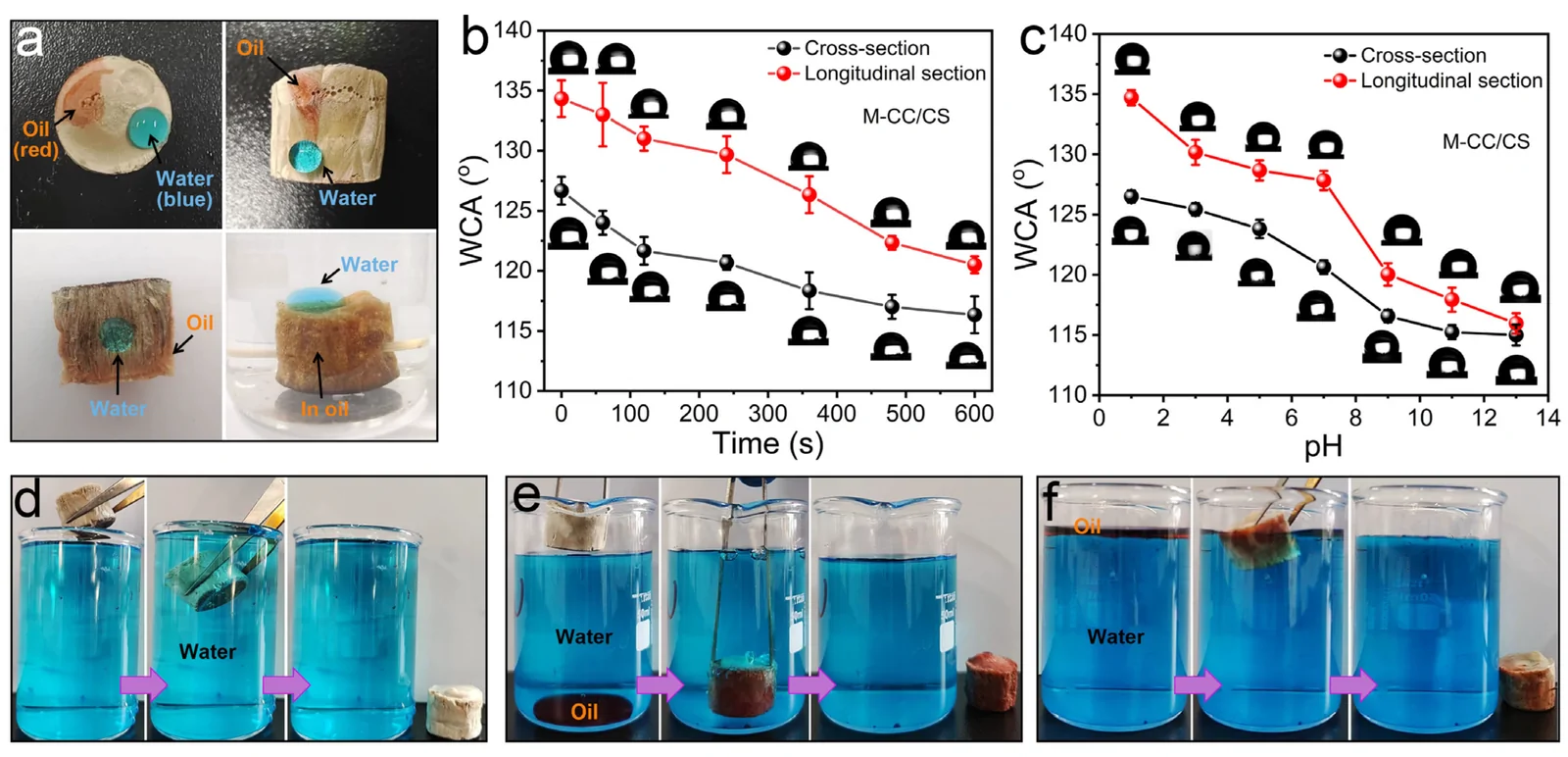
(**a**) Photographs of water and oil droplets on M-CC/CS, including a water droplet under oil; (**b**) WCA stability on the longitudinal and cross-sectional surfaces; (**c**) WCA at different pH values; (**d**) immersion of M-CC/CS in dyed water; and selective absorption of (**e**) dense oil beneath water and (**f**) light oil floating on water.

After MTMS vapor treatment, M-CC/CS exhibited hydrophobic and oleophilic behavior. Water droplets remained approximately spherical on the external and internal surfaces, whereas oil droplets were rapidly absorbed (Figure 7a). A water droplet also remained stable on the aerogel in an oil environment, demonstrating under-oil water repellency. The water contact angles on both the longitudinal and cross-sectional surfaces decreased gradually with contact time but remained above 115° after 600 s (Figure 7b). Under different pH conditions, the longitudinal surface consistently exhibited a higher contact angle than the cross-section (Figure 7c). This difference is attributed to the aligned layered structure on the longitudinal surface, which forms stable air pockets to strengthen hydrophobicity, while the honeycomb through-pores on the cross-section expose more polar residual groups, reducing water repellency. The contact angle decreased at high pH, possibly because residual polar groups became more exposed or ionized. M-CC/CS selectively removed both a dense oil phase beneath water and a light oil phase floating on water (Figure 7d–f, Videos S2–S4), confirming its applicability to free-oil capture.

**Figure 8:**
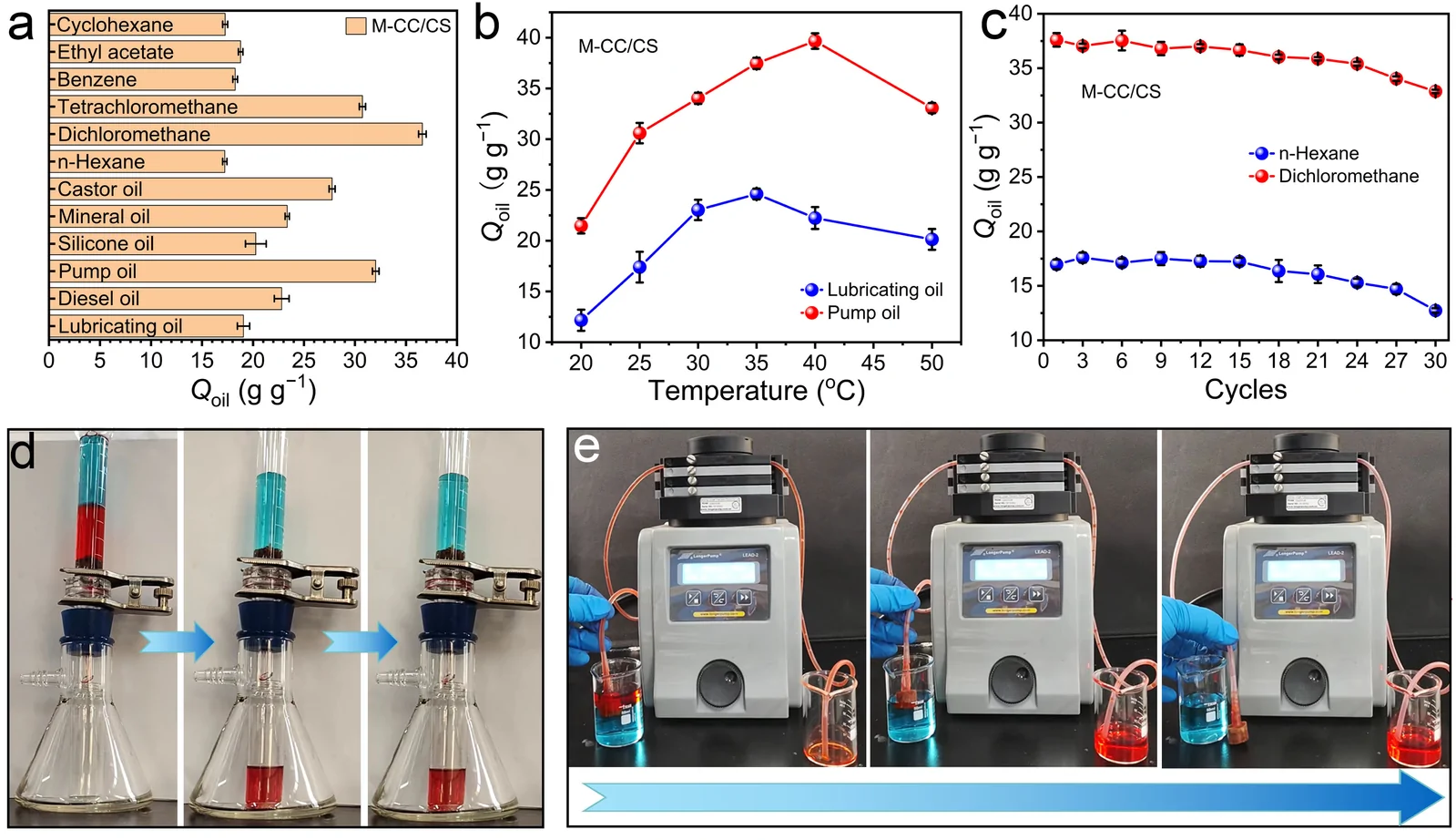
Oil-absorption and oil/water mixture separation performance of M-CC/CS: (**a**) absorption capacities for oils and organic solvents; (**b**) absorption capacities of pump oil and lubricating oil at different temperatures; (**c**) cyclic absorption of n-hexane and dichloromethane; (**d**) gravity-driven separation of an immiscible oil/water mixture; and (**e**) continuous oil/water mixture separation.

M-CC/CS absorbed a broad range of oils and organic solvents, with capacities of 16.22–40.13 g g^−1^ (Figure 8a). The differences in uptake reflect the combined effects of liquid density, viscosity, surface affinity, and retention within the pore volume. For pump oil and lubricating oil, the absorption capacity first increased and then decreased as the temperature rose (Figure 8b). Moderate heating reduced oil viscosity and accelerated liquid penetration into three-dimensional pore networks, raising saturated absorption capacity. Excessively high temperature weakens oil intermolecular forces, leading to oil outflow from aerogel pores and decreased absorption performance. During 30 absorption–desorption cycles with n-hexane and dichloromethane, the capacities decreased gradually but remained appreciable (Figure 8c), demonstrating reusable absorption performance rather than complete capacity retention. Hornification associated with repeated drying may also contribute to this decline by promoting irreversible cellulose microfibril association and partial pore contraction, although this effect was not directly characterized in the present study [32]. For an immiscible oil/water mixture, the oil phase passed through M-CC/CS under gravity while water was retained above the aerogel (Figure 8d). The measured separation flux reached 565.47 L m^−2^ h^−1^. The same wetting selectivity enabled continuous separation using a peristaltic-pump-assisted setup (Figure 8e). The overall absorption and separation performances are comparable with representative cellulose-based aerogels.

### 2.4. W/O Emulsion Separation

**Figure 9:**
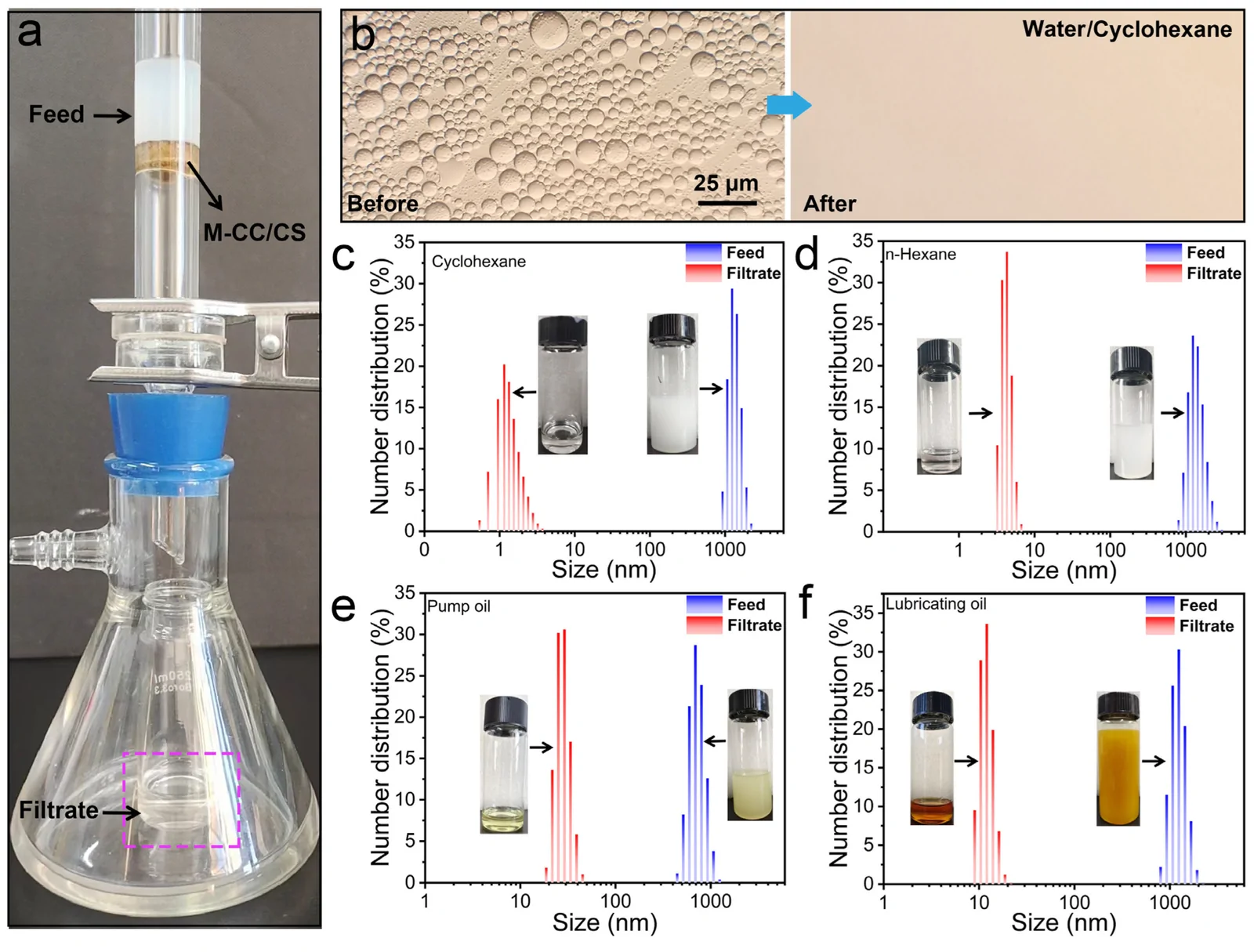
(**a**) Gravity-driven W/O emulsion separation device containing M-CC/CS; (**b**) optical microscopy images of the water/cyclohexane emulsion before and after separation; and droplet-size distributions and photographs of the feed and filtrate for (**c**) water/cyclohexane, (**d**) water/n-hexane, (**e**) water/pump oil, and (**f**) water/lubricating oil emulsions.

**Figure 10:**
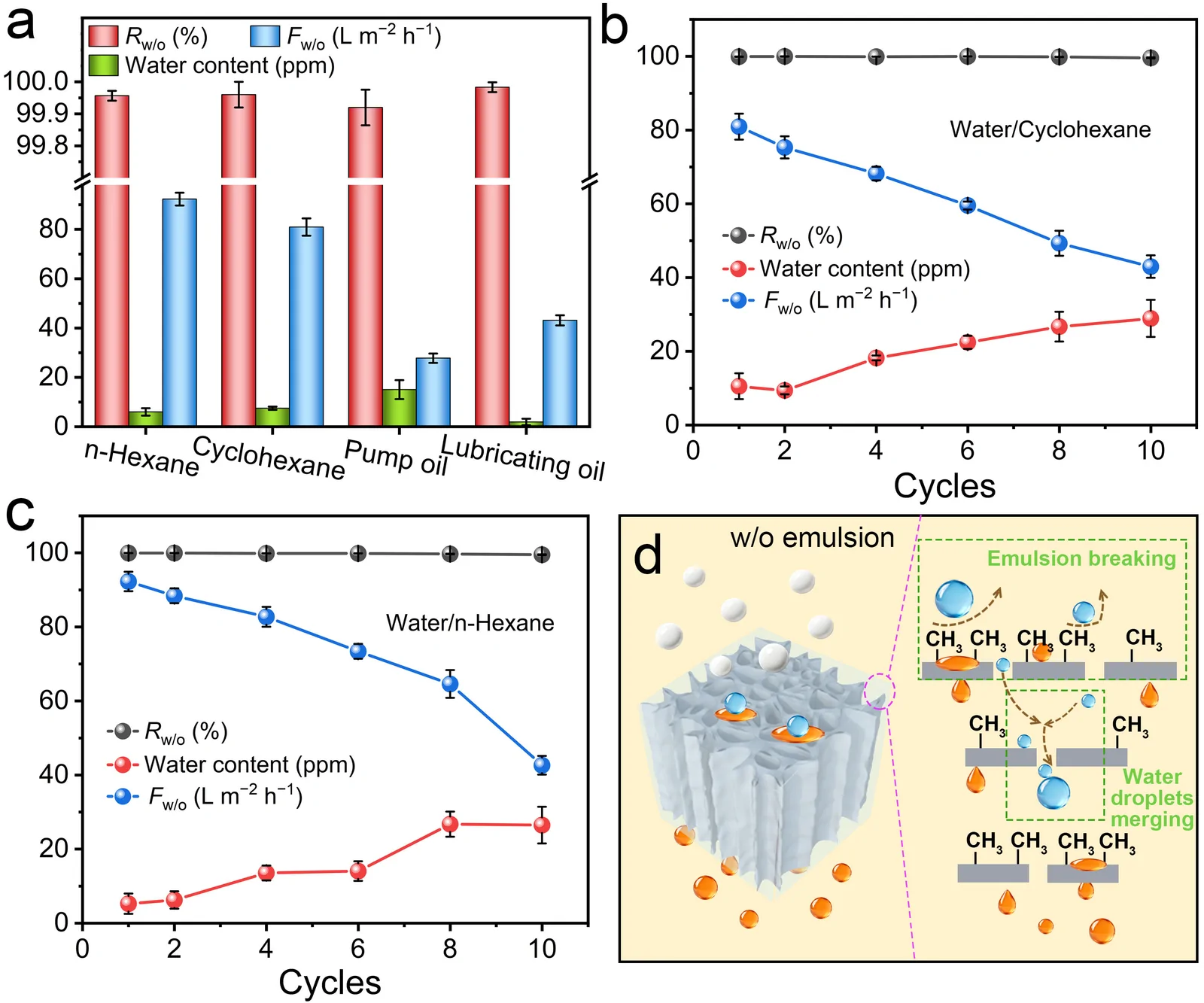
(**a**) Separation efficiency, water content, and flux for four W/O emulsions; cyclic separation of (**b**) water/cyclohexane and (**c**) water/n-hexane emulsions; and (**d**) schematic illustration of the W/O emulsion separation mechanism of M-CC/CS.

M-CC/CS was further evaluated for the gravity-driven separation of W/O emulsions. In the separation device, the emulsion contacted the aerogel in a glass tube and the oil-rich filtrate was collected below (Figure 9a). After passage through M-CC/CS, the initially turbid water/cyclohexane emulsion became optically transparent, and no micrometer-scale droplets were observed in the filtrate (Figure 9b). Similar behavior was observed for water/n-hexane, water/pump-oil, and water/lubricating-oil emulsions. The apparent droplet-size distributions shifted from approximately 800–3000 nm in the feeds to below approximately 20 nm in the filtrates (Figure 9c–f). For all four emulsions, the measured water-removal efficiencies exceeded 99.9%, the filtrate water contents were below 20 ppm, and the fluxes ranged from 25.68 to 91.21 L m^−2^ h^−1^ (Figure 10a). The lower fluxes of pump-oil and lubricating-oil emulsions are attributed to residual micron-sized water droplets and emulsifier Span 80 adsorbed inside tortuous pore channels, which increase mass transfer resistance; meanwhile, long-term immersion in the oil phase causes slight hydrolysis of the MTMS silane coating, reducing the local oleophilicity of the pore inner walls.

The cyclic separation performance was evaluated using water/cyclohexane and water/n-hexane emulsions (Figure 10b,c). The separation efficiency remained above 99% after 10 cycles, and the filtrate water content remained below 35 ppm. The flux decreased progressively with cycle number, particularly for the water/n-hexane emulsion. The decrease is attributed to residual water droplets and trace contaminants retained within the pore network, which increase transport resistance. The proposed separation mechanism is illustrated in Figure 10d. When w/o emulsion moves to the surface of the material under the action of gravity, large-size emulsion droplets will be demulsified under the porous and rough interface, and the initial separation of oil and water will be realized. Because of the super-oleophilic and hydrophobic properties of M-CC/CS, the oil droplets will penetrate through the material and be blocked on its surface. The small-sized emulsion will continue to go down and break the emulsion on the smaller three-dimensional pore structure to separate. Under the push of gravity, small water droplets in the material converge and form large water droplets larger than the pore size, which are then blocked in the material, thus realizing the separation of water/oil emulsion.

As summarized in Table 1, CC/CS exhibited competitive CR adsorption (483.09 mg g^−1^, 95.67%), while M-CC/CS achieved an oil/organic-liquid absorption capacity of 16.22–40.13 g g^−1^ and a W/O emulsion separation efficiency above 99.9%. These results demonstrate the complementary applicability of the cotton-stalk-derived aerogel system to dye adsorption and W/O emulsion separation.

## 3. Conclusions

An anisotropic cotton stalk-derived CC/CS aerogel was prepared via lignocellulose purification, chitosan-assisted hydrothermal treatment, glutaraldehyde crosslinking, and unidirectional freeze-drying. The hydrophilic CC/CS aerogel combined a low density, high porosity, and abundant oxygen- and nitrogen-containing groups, enabling effective CR adsorption. The equilibrium data were described well by the pseudo-second-order kinetic and Langmuir isotherm models, with a calculated maximum capacity of 483.09 mg g^−1^. Adsorption was favored under acidic conditions and was governed by combined electrostatic, hydrogen-bonding, and pore-transport effects. The material tolerated NaCl but was more strongly affected by cationic surfactants, and its adsorption performance decreased over five regeneration cycles. MTMS-modified M-CC/CS exhibited hydrophobic/oleophilic behavior, absorbed oils and organic solvents at 16.22–40.13 g g^−1^, and enabled gravity-driven separation of immiscible oil/water mixtures at 565.47 L m^−2^ h^−1^. For several W/O emulsions, the separation efficiency exceeded 99.9% and remained above 99% after 10 cycles, although the flux gradually decreased. These results demonstrate complementary use of the hydrophilic and hydrophobically modified forms of a single biomass-derived aerogel platform for dye adsorption and oily water treatment.

## 4. Materials and Methods

### 4.1. Materials

Cotton stalks were collected from Farm 12 in Aral City, Xinjiang, China. Sodium chlorite, anhydrous ethanol, sodium chloride (NaCl), and glacial acetic acid were purchased from Tianjin Zhiyuan Chemical Reagents Co., Ltd. (Tianjin, China). Formic acid was obtained from Tianjin Xinbote Chemical Co., Ltd. (Tianjin, China) CS (degree of deacetylation: 80.0–95.0%; viscosity: 50–800 mPa·s) was purchased from Shanghai Aladdin Biochemical Technology Co., Ltd. (Shanghai, China) and glutaraldehyde was obtained from Tianjin Kemio Chemical Reagents Co., Ltd. (Tianjin, China) Zinc acetate was purchased from Tianjin Beichen Huayue Chemical Reagent Factory (Tianjin, China). MTMS was supplied by Shanghai Macklin Biochemical Technology Co., Ltd. (Shanghai, China) CTAB, SDBS, CR, sulfuric acid, n-hexane, carbon tetrachloride, lubricating oil, diesel, and Span 80 were purchased from Aladdin Co., Ltd. (Shanghai, China) and used as received. Cyclohexane, pump oil, and the other oils and organic solvents used in the absorption tests were commercially obtained and used without further purification.

### 4.2. Preparation of CC

Crushed cotton stalk powder (5 g) was dispersed in 60 mL of formic acid and refluxed in an oil bath at 90 °C for 3 h to disrupt the compact lignocellulosic structure and partially remove hemicellulose and lignin, thereby facilitating subsequent cellulose purification. The solid was washed repeatedly with deionized water to neutral pH and dried at 75 °C to obtain intermediate A. Intermediate A (2 g) was dispersed in 75 mL of deionized water, followed by the addition of 1 g of sodium chlorite and 0.5 mL of glacial acetic acid. Sodium chlorite was used for further oxidative removal of residual lignin, while glacial acetic acid provided the acidic conditions required for effective delignification. The suspension was stirred at 75 °C for 1 h, washed with deionized water, and subjected to the same delignification treatment three times. The purified solid was dried at 75 °C to obtain intermediate B. Intermediate B (1 g) was dispersed in 60 g of 64 wt.% H_2_SO_4_ and stirred at room temperature for 12 h to preferentially hydrolyze the amorphous regions of cellulose while retaining the more ordered crystalline regions. The suspension was centrifuged, washed three times with deionized water, and freeze-dried to obtain cellulose. A chitosan solution was prepared by dissolving 1 g of chitosan in 100 mL of deionized water containing 1 mL of acetic acid. A cellulose suspension was prepared by dispersing 1 g of cellulose in 100 mL of deionized water and stirring for 1 h. Equal volumes (15 mL each) of the chitosan solution and cellulose suspension were mixed and hydrothermally treated at 180 °C for 10 h. The product was washed three times with deionized water and dried at 75 °C. The resultant chitosan-modified cellulose composite was designated CC.

### 4.3. Preparation of CC/CS Aerogels

**Figure 11:**
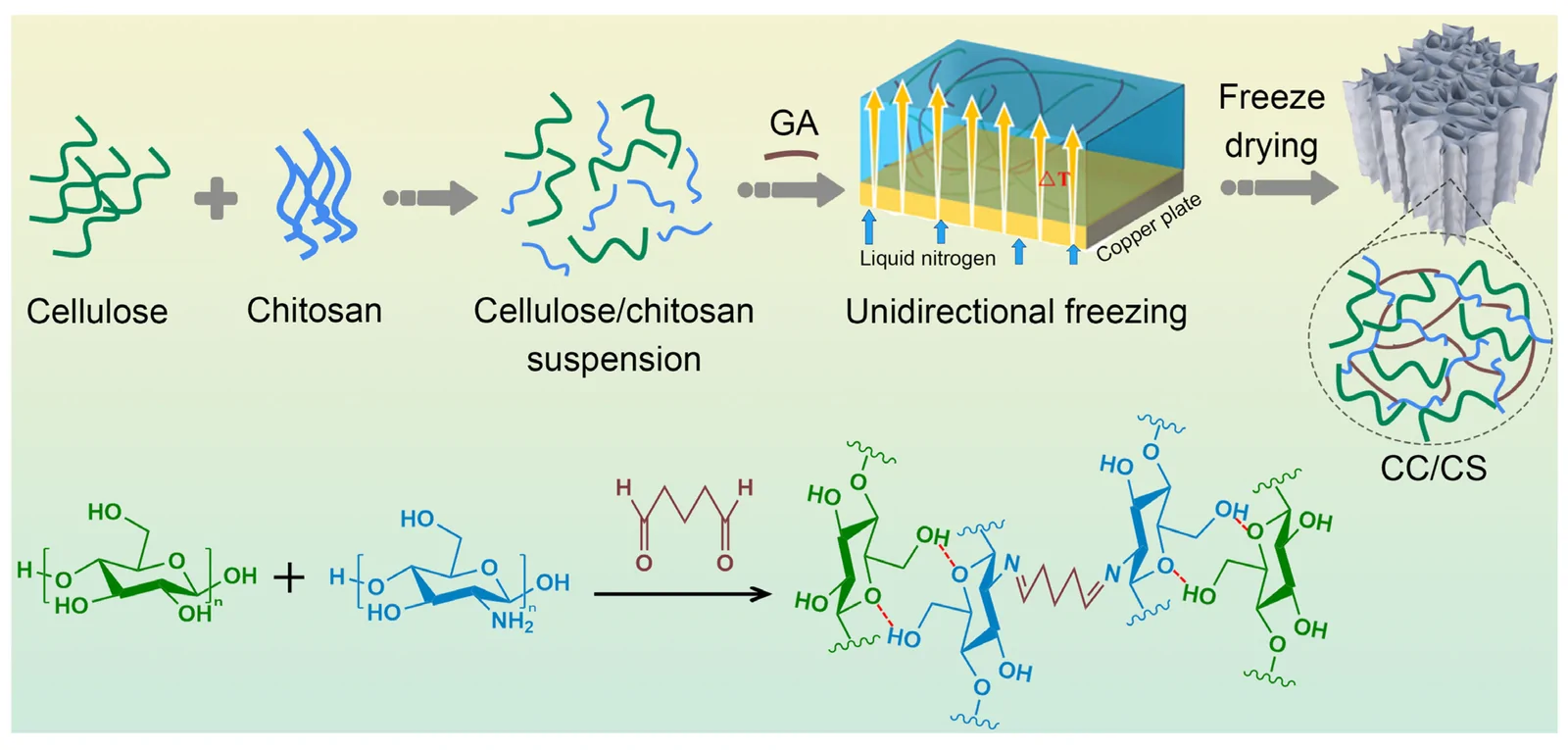
Schematic illustration of the preparation of CC/CS aerogels.

A 1 wt.% CC suspension was prepared by dispersing 1 g of CC in 100 mL of deionized water, followed by stirring for 1 h and sonication for 30 min. A 1.5 wt.% CS solution was prepared by dissolving 1.5 g of CS and 0.3 g of zinc acetate in 100 mL of deionized water containing 1 mL of acetic acid. Zinc acetate was introduced as an auxiliary network-stabilizing component, with Zn^2+^ potentially interacting with the amino and hydroxyl groups of chitosan and the hydroxyl groups of cellulose through coordination interactions. The CC suspension (20 mL) was mixed with an equal volume of the CS solution and stirred for 10 min. Glutaraldehyde (400 μL) was then added, and the mixture was stirred for a further 30 min at room temperature without additional heating. The resulting suspension was poured into a mold and directionally frozen using liquid nitrogen, followed by storage under frozen conditions for 12 h. The frozen sample was freeze-dried at −55 °C for 48 h and subsequently heated at 75 °C for 1 h to further promote the reaction between the aldehyde groups of glutaraldehyde and the amino groups of chitosan, thereby enhancing the formation of the crosslinked network and yielding the CC/CS aerogel (Figure 11).

### 4.4. Preparation of M-CC/CS Aerogels

Dry CC/CS monoliths were placed in a sealed 500 mL glass reactor with 2 mL pure MTMS liquid at the bottom (no direct contact between aerogel and liquid MTMS). The sealed vessel was heated to 75 °C in an oven for 8 h to realize saturated MTMS vapor modification. After cooling to room temperature, the aerogels were placed in a fume hood for 2 h to volatilize residual unreacted MTMS vapor, and then named M-CC/CS.

### 4.5. Characterization

Fourier transform infrared (FTIR) spectra were recorded using a Bruker Vertex 70 (Billerica, MA, USA) spectrometer over 400–4000 cm^−1^. An X-ray diffractometer (XRD; BrukerAXS D8, Madison, MA, USA) was used to analyze the crystal form of the samples. Thermogravimetric analysis (TA Instruments SDT Q600, New Castle, DE, USA) was performed under nitrogen from 25 to 750 °C at a heating rate of 10 °C min^−1^. The microstructures were observed via scanning electron microscopy (SEM; ZEISS Sigma 300, Baden-Württemberg, Germany). Static water contact angles were measured at room temperature using a JJ2000B contact-angle goniometer (Shanghai Zhongchen Digital‑Technology Equipment Co., Ltd., Shanghai, China) with 4 μL water droplets. Timing was started when the droplet contacted the sample surface and detached from the dispensing needle, which was defined as t = 0 s. Zeta potentials and emulsion droplet-size distributions were measured using a Malvern Nano ZS90 analyzer (Worcestershire, UK). The water content of the feed and filtrate was measured by Karl Fischer titration using an HD-WS2 moisture analyzer (Shandong Horde Electronic Technology Co., Ltd., Jinan, China). CR concentrations were determined at 500 nm using a UV–visible spectrophotometer (UV1901PCS, Shanghai Youke, Shanghai, China).

### 4.6. Density, Porosity, and Water Absorption

The bulk density (*ρ*, g cm^−3^) and porosity (*P*, %) were determined using the geometric-volume and ethanol-displacement methods and calculated using Equations (1) and (2), respectively.

(1) $$\rho =\frac{m_{0}}{V}$$

(2) $$P(\%)=\frac{M_{1}-m_{0}}{\rho_{s}V}\times 100\%$$

where *m*_0_ is the dry mass of the sample (g), *V* is its apparent geometric volume (cm^3^), *M*_1_ is the total mass after complete ethanol infiltration (g), and *ρ*_s_ is the density of anhydrous ethanol (0.789 g cm^−3^). The water-absorption capacity was determined using a gravimetric saturation method. The dry aerogel was weighed, saturated with water, and reweighed. The water-absorption capacity was calculated from the mass increase relative to the dry mass and was expressed as grams of water per gram of dry aerogel. All experiments were performed with three parallel replicates.

### 4.7. Adsorption Experiments

CR was used as a model anionic dye. CC/CS (10 mg) was added to 10 mL of CR solution and agitated in a thermostatic shaker at 298 K and 150 rpm. The effects of pH, contact time, initial CR concentration, and coexisting species were evaluated. For the pH experiments, the initial pH was adjusted over the range shown in Figure 3. Kinetic experiments were conducted for up to 600 min, and isotherm experiments were performed using initial CR concentrations up to 1000 mg L^−1^. NaCl, CTAB, and SDBS were added at the concentrations indicated in Figure 3d–f to evaluate interference effects. After adsorption, the supernatant was separated, diluted when necessary, and analyzed at 500 nm. The equilibrium adsorption capacity and removal efficiency were calculated using Equations (3) and (4) [37].

(3) $$Q_{e}=\frac{C_{0}-C_{e}}{m}\times V$$

(4) $$R=\frac{C_{i,0}-C_{i,f}}{C_{i,0}}\times 100\%$$

where *Q*_e_ is the equilibrium adsorption capacity (mg g^−1^), *C*_0_ is the initial CR concentration (mg L^−1^), and *C*_e_ is the equilibrium *C*_R_ concentration (mg L^−1^). *C*_i,0_ and *C*_i,f_ represent the concentrations of contaminants (CR dye and W/O emulsion) in the feed and filtrate, respectively. *V* is the solution volume (L), and *m* is the mass of CC/CS (g).

The kinetic data were fitted using the pseudo-first-order model (Equation (5)), the pseudo-second-order model (Equation (6)), and the intraparticle-diffusion model (Equation (7)). The equilibrium data were fitted using the Freundlich model (Equation (8)) and Langmuir model (Equation (9)) [38].

(5) $$\ln(Q_{e}-Q_{t})=\ln Q_{e}-K_{1}t$$

(6) $$\frac{t}{Q_{t}}=\frac{1}{K_{2}Q_{e}^{2}}+\frac{t}{Q_{e}}$$

(7) $$Q_{t}=K_{i}t^{0.5}+C$$

(8) $$\mathrm{lg}Q_{e}=\mathrm{lg}K_{F}+\frac{1}{n}\mathrm{lg}C_{e}$$

(9) $$\frac{C_{e}}{Q_{e}}=\frac{1}{K_{L}Q_{\max}}+\frac{C_{e}}{Q_{\max}}$$

where *Q*_t_ is the adsorption capacity at time *t* (mg g^−1^), *K*_1_ is the pseudo-first-order rate constant (min^−1^), *K*_2_ is the pseudo-second-order rate constant (g mg^−1^ min^−1^), *K*_i_ is the intraparticle diffusion rate constant (mg g^−1^ min^−0^.^5^), and *C* is the boundary-layer intercept (mg g^−1^). *Q*_max_ is the Langmuir maximum adsorption capacity (mg g^−1^), *K*_L_ is the Langmuir constant, *K*_F_ and n are the Freundlich constants, and *C*_e_ is the equilibrium *C*_R_ concentration (mg L^−1^).

For the regeneration experiments, CC/CS (10 mg) was added to 10 mL of a 500 mg L^−1^ CR solution and agitated at 298 K and 150 rpm until adsorption equilibrium was reached. The CR-loaded adsorbent was subsequently recovered via centrifugation and transferred into 100 mL of absolute ethanol. Desorption was performed at 25 °C under continuous stirring. The adsorbent was recovered after 4 h, and the spent ethanol was replaced with 100 mL of fresh absolute ethanol. This desorption procedure was repeated three times, resulting in a total desorption time of 12 h. After the final desorption step, the regenerated adsorbent was recovered and reused in a fresh 500 mg L^−1^ CR solution under the same adsorption conditions. A total of five consecutive adsorption–desorption cycles were conducted.

### 4.8. Oil Absorption and Oil/Water Separation Tests

For oil-absorption tests, a dry M-CC/CS sample was weighed, immersed in an oil or organic solvent until saturation, removed, and allowed to drain before reweighing. The saturated absorption capacity was calculated using Equation (10). Cyclic absorption tests were conducted with n-hexane and dichloromethane for 30 cycles; the absorbed liquid was removed between successive cycles before the aerogel was reused. Immiscible oil/water mixtures were prepared using equal volumes of oil and water and were separated under gravity through an M-CC/CS aerogel mounted in a filtration device. Continuous separation was evaluated using a peristaltic pump. W/O emulsions were prepared by mixing 99 mL of oil phase (n-hexane, cyclohexane, pump oil, or lubricating oil) with 1 mL of deionized water and 0.25 g of Span 80, followed by magnetic stirring for 3 h. The emulsions were passed through M-CC/CS under gravity. Separation performance was evaluated from the visual appearance, droplet-size distribution, Karl Fischer water content, water-removal efficiency, and flux. The oil-absorption capacity (Equation (10)) and flux (Equation (11)) were calculated as follows:

(10) $$Q_{\mathrm{oil}}=\frac{\left(m_{1}-m_{0}\right)}{m_{0}}$$

(11) $$F=\frac{4V}{\pi d^{2}\Delta t}$$

where *Q*_oil_ is the saturated oil-absorption capacity (g g^−1^), *m*_0_ and *m*_1_ are the masses of the dry and oil-saturated aerogels (g), respectively, *F* is the separation flux (L m^−2^ h^−1^), *V* is the filtrate volume (L), *d* is the effective diameter of the separation area (m), and Δ*t* is the filtration time (h).

## Figures and Tables

**Table 1:** Comparison of representative cellulose-based aerogels.

Aerogel	Application	*Q*_CR_ (mg g^−1^)	*R*_CR_ (%)	*Q*_Oil_ (g g^−1^)	R_W/O_ (%)	Ref.
3D Cellulose/MoS_2_	CR adsorption	334.92	NR	NR	NR	[33]
Biohybrid aerogel	CR adsorption	559.6	94.7	NR	NR	[34]
Cellulose	Oil absorption, W/O separation	NR	NR	5–125	97.8–99.1	[35]
Bio-inspired castor oil modified cellulose	Oil absorption, W/O separation	NR	NR	53.2–113.8	94.4–97.1	[36]
CC/CS	CR adsorption	483.09	95.67	NR	NR	This work
M-CC/CS	Oil absorption, W/O separation	NR	NR	16.22–40.13	>99.9	

NR: Not reported in the corresponding reference. Values reported in different studies were obtained under different experimental conditions and are provided for general comparison only.

## Data Availability

The data presented in this study are available from the corresponding author upon reasonable request.

## Supplementary Materials

The following supporting information can be downloaded at https://www.mdpi.com/article/10.3390/gels12090814/s1. Video S1: Compression and recovery of cc/cs. Video S2: Immersion of M-CC/CS in dyed Water. Video S3: M-CC/CS selectively adsorbs underwater heavy oil. Video S4: M-CC/CS selectively adsorbs light oil on water.
